# Dynamic, Interpretable, Machine Learning–Based Outcome Prediction as a New Emerging Opportunity in Acute Ischemic Stroke Patient Care: A Proof-of-Concept Study

**DOI:** 10.1155/srat/3561616

**Published:** 2025-03-25

**Authors:** Ivan Petrović, Sava Njegovan, Olivera Tomašević, Dmitar Vlahović, Sonja Rajić, Željko Živanović, Isidora Milosavljević, Ana Balenović, Nikola Jorgovanović

**Affiliations:** ^1^Faculty of Medicine, University of Novi Sad, Novi Sad, Serbia; ^2^Department of Computing and Control Engineering, Faculty of Technical Sciences, University of Novi Sad, Novi Sad, Serbia; ^3^Department of Neurology, Faculty of Medicine, University of Novi Sad, Novi Sad, Serbia; ^4^Neurology Clinic, University Clinical Center of Vojvodina, Novi Sad, Serbia

## Abstract

**Introduction:** While the machine learning (ML) model's black-box nature presents a significant barrier to effective clinical application, the dynamic nature of stroke patients' recovery further undermines the reliability of established predictive scores and models, making them less suitable for accurate prediction and appropriate patient care. This research is aimed at building and evaluating an interpretable ML-based model, which would perform outcome prediction at different time points of patients' recovery, giving more secure and understandable output through interpretable packages.

**Materials and Methods:** A retrospective analysis was conducted on acute ischemic stroke (AIS) patients treated with alteplase at the Neurology Clinic of the University Clinical Center of Vojvodina (Novi Sad, Serbia), for 14 years. Clinical data were grouped into four categories based on collection time—baseline, 2-h, 24-h, and discharge features—serving as inputs for three different classifiers—support vector machine (SVM), logistic regression (LR), and random forest (RF). The 90-day modified Rankin scale (mRS) was used as the outcome measure, distinguishing between favorable (mRS ≤ 2) and unfavorable outcomes (mRS ≥ 3).

**Results:** The sample was described with 49 features and included 355 patients, with a median age of 67 years (interquartile range (IQR) 60–74 years), 66% being male. The models achieved strong discrimination in the testing set, with area under the curve (AUC) values ranging from 0.80 to 0.96. Additionally, they were compared with a model based on the DRAGON score, which showed an AUC of 0.760 (95% confidence interval (CI), 0.640–0.862). The decision-making process was more thoroughly understood using interpretable packages: Shapley additive explanation (SHAP) and local interpretable model–agnostic explanation (LIME). They revealed the most significant features at both the group and individual patient levels.

**Conclusions and Clinical Implications:** This study demonstrated the moderate to strong efficacy of interpretable ML-based models in predicting the functional outcomes of alteplase-treated AIS patients. In all constructed models, age, onset-to-treatment time, and platelet count were recognized as the important predictors, followed by clinical parameters measured at different time points, such as the National Institutes of Health Stroke Scale (NIHSS) and systolic and diastolic blood pressure values. The dynamic approach, coupled with interpretable models, can aid in providing insights into the potential factors that could be modified and thus contribute to a better outcome.

## 1. Introduction

Even though the acute treatment of ischemic stroke has been significantly improved, this condition still represents one of the leading causes of mortality, hospitalization, and long-term disability [1–7]. The complex and dynamic nature of stroke recovery has been previously investigated, and it is established that the functional outcome depends on both baseline characteristics and poststroke complications [8, 9]. In the currently available literature, several predictive scores and multivariable predictive models have been constructed and validated for this matter [10]. However, most of these utilize only one point in time for prediction, devaluating stroke recovery's dynamic and variable nature.

Dynamic prediction provides an estimate of future outcomes at specific time points, based on all available patient data up to that time [11], and in currently available literature, it is evident that the quantity of papers utilizing this technology in stroke patients is comparatively small. The term itself is ambiguous, as it could imply a time series network, which could reveal complicated behaviors from time series [12]. However, in this research, as well as in some previous studies [13], the term refers to making predictions at multiple time points, rather than monitoring the dynamics of parameter value changes. As a dynamic approach could result in increased data size and higher computational expenses, machine learning (ML) represents a great candidate for this task, as it demonstrated significant worth as an ultimate technology in helping health professionals make clinical decisions and, as previously proven, outperforms standard statistical approaches [14–18]. Some of the previous research that utilized the dynamic approach in acute ischemic stroke (AIS) patients exploited the power of ML models. They have analyzed the potential of a dynamic prediction in AIS patients treated with both intravenous thrombolysis (IVT) and mechanical thrombectomy (MT) at various time points, five and four, respectively, and both approaches demonstrated higher predictive power compared to the well-known predictive scores [13, 19]. Although these results have shown that the dynamic approach done with ML models could be the new prediction standard, there is still a missing link that would enable using these models in everyday practice. Due to the ML model's complexity, the inner logic and prediction-making process are not readily intelligible and understandable to a human, labeling this problem as a black box of a model [20, 21]. To implement models in a real clinical environment, clinicians should be aware of why and how decisions are being made, and a high level of transparency regarding the decision-making process should be provided [22, 23]. Fortunately, this problem has been solved by the development of interpretable frameworks, such as Shapley additive explanations (SHAPs) and local interpretable model–agnostic explanations (LIMEs), additionally facilitating model usage in both research and a clinical environment [24, 25].

In order to gain a better understanding and identify gaps in the current literature, we present some of the previously developed ML- and deep learning (DL)–based models for predicting outcomes in AIS patients (Table 1). It is noticeable that the majority of previous studies did not incorporate a multi–time point approach. However, in the studies where this approach was utilized, later time points consistently demonstrated higher AUC values. Among studies that employed interpretable tools to understand the decision-making process of a model, feature-level interpretation with SHAP was predominantly used. While SHAP helps to understand the impact of features on an outcome and their collinearity, LIME offers an advantage for clinicians by making the results more easily interpretable on an individual patient level, enabling a clearer understanding of how predictions are made for real-life scenarios.

Since previously described dynamic models could be improved, this research is aimed at constructing and validating dynamic, interpretable ML-based models for alteplase-treated AIS patients, which would demonstrate the real significance of a multi–time point prediction process, as well as its interpretation. These newly constructed models could potentially represent a new state-of-the-art approach to the prediction of ischemic stroke patients' outcomes.

## 2. Materials and Methods

### 2.1. The Sample Formation

This retrospective study analyzed data of AIS patients, treated with intravenous alteplase, at the Neurology Clinic of the University Clinical Center of Vojvodina (Novi Sad, Serbia), during a 14-year period (2008–2022). The study was approved by the Local Ethics Committee (Approval Number 00-4, Date: 13 January 2023). The data were collected from the Clinical Information System, and the inclusion criteria were as follows: (I) A patient was older than 18 years; (II) the patient had no history of stroke; (III) intravenous alteplase was used as a treatment option; and (IV) a 90-day functional outcome, expressed as a modified Rankin scale (mRS) score, was known. On the contrary, ischemic stroke patients who were not treated with alteplase, or without known outcomes, were excluded from the study.

### 2.2. Statistical Analysis

The division into the outcome groups was made by the value of the 90-day mRS, and the groups were as follows: (I) patients with favorable outcomes (mRS ≤ 2) and (II) patients with unfavorable outcomes (mRS ≥ 3). The previously described division was adopted to maintain feasibility and align with the methodology of earlier research involving AIS patients (see Table 1).

Data processing was carried out in Python ver. 3.10.6 (Python Software Foundation, Wilmington, Delaware, United States) [35]. The first step was screening variables for missing values and excluding them from further analysis if this number exceeded 20%. Categorization of the variables followed, in which the division was made into categorical and continuous groups. During imputation, the most common value method was used for categorical variables, while the median value was used for continuous ones. Forty-nine variables were analyzed using adequate tests. Categorical variables were expressed as numbers (percentages), and the chi-squared test was used to determine differences between the two groups. Continuous variables were presented as medians (interquartile range) and analyzed by Student *t*-test or Mann–Whitney *U* test, based on sample normality. Two-tailed tests were used, and statistical significance was observed at level *p* < 0.05, for every variable. For continuous data normalization, *Z*-score was used to reduce numerical instabilities between the analyzed features [36].

### 2.3. ML Model Build-Up

**Figure 1:**
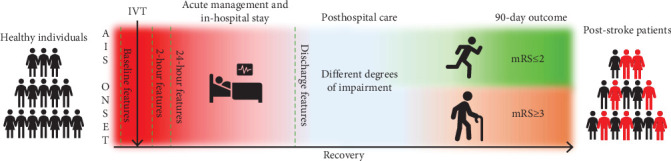
The data were collected at four different time points, and the outcome was estimated as a 90-day mRS value. AIS: acute ischemic stroke, IVT: intravenous thrombolytic therapy, mRS: modified Rankin score.

**Figure 2:**
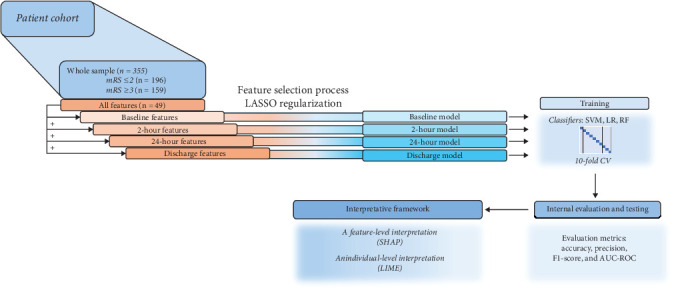
A summary of the research steps, which included data preprocessing, model training, and internal evaluation, as well as visualizing the decision-making process is presented in the figure. mRS: modified Rankin score, LASSO: Least Absolute Selection and Shrinkage Operator, SVM: support vector machine, LR: logistic regression, RF: random forest, CV: cross-validation, AUC-ROC: the area under the curve of the receiver operating characteristic, SHAP: Shapley additive explanations, LIME: local interpretable model–agnostic explanations.

The sample was described with 49 features. Based on the time point at which they were collected, the features defined four datasets: (I) baseline data, (II) 2-h data, (III) 24-h data, and (IV) discharge data (Figure 1). Based on this division, models encompassing different time frames were constructed: (I) The baseline model included baseline data; (II) the 2-h model included baseline and 2-h data, (III) the 24-h model included baseline, 2-h, and 24-h data; and (IV) the discharge model included all four datasets. The whole process and the research pipeline are summarized in Figures 1 and 2.

The dataset was split into a training set (80%) and a testing set (20%), by random splitting. The testing set was used to evaluate model performance. The training set was used for the feature selection process, which was carried out using the Least Absolute Selection and Shrinkage Operator (LASSO). This method is a regression analysis algorithm, which is often used to minimize potential collinearity, reduce a high-dimensional feature space by excluding noninterference variables, and reduce the overfitting of variables, which improves both prediction accuracy and interpretability [24, 37, 38]. The regularization parameter (alpha) was set at 0.2. Following the methodology of another study [13], 10 features with the highest importance were selected and used for the model training for every time point. The time point–based division of features along with their feature (patient data, clinical data, laboratory data, neuroradiological assessment, vascular risk factors, treatment data, stroke data, outcomes, discharge data, and hospitalization data) and variable (categorical or continuous) types is shown in Table 2. Features' description is presented in Table S1.

#### 2.3.1. Classifiers

Even though there is no single answer when it comes to classifier selection, appropriate models should be employed according to the characteristics of data and research purpose, while the results can vary depending on the sample size. In our research, at each time point, three classifiers were used: support vector machine (SVM), logistic regression (LR), and random forest (RF), and they were trained and evaluated on datasets made upon selected features. SVM and LR are both linear classifiers, where the first one is aimed at finding the optimal hyperplane that maximizes the margin between the classes, and the second one uses logistic function [39]. RF is an ensemble method that constructs multiple decision trees, which makes it a nonlinear classifier and by that capable of capturing complex interactions between variables. In previous research, LR has shown superiority in terms of prediction accuracy when it comes to smaller sample sizes, while the predictive power of ensemble classifiers, such as RF, improves with an increase in sample size [40]. In order to optimize the hyperparameters of all the classifiers, the grid search was employed. It evaluated multiple configurations of the model's parameters using 10-fold cross-validation (CV) and selected the combination that achieved the best performance, based on accuracy. The chosen parameters were then used for further analysis and reporting.

### 2.4. Models' Evaluation

The evaluation metrics used were accuracy, precision, sensitivity, F1 score, and area under the curve (AUC) of the receiver operating characteristic (AUC-ROC). The previously mentioned parameters were calculated using the following formulas [41, 42]:

(1) $$\begin{array}{c} \text{Accuracy}=\frac{\text{true positives}+\text{true negatives}}{\text{true positives}+\text{true negatives}+\text{false positives}+\text{false negatives}} \end{array}$$

(2) $$\begin{array}{c} \text{Precision}=\frac{\text{true positives}}{\text{true positives}+\text{false positives}} \end{array}$$

(3) $$\begin{array}{c} \text{Sensitivity}=\frac{\text{true positives}}{\text{true positives}+\text{false negatives}} \end{array}$$

(4) $$\begin{array}{c} \text{F}1\text{ score}=2*\frac{\text{precision}*\text{sensitivity}}{\text{precision}+\text{sensitivity}} \end{array}$$

The AUC measures the ability of a classifier to distinguish between classes. It is derived from the receiver operating characteristic (ROC) curve, which plots the true positive rate (TPR) against the false positive rate (FPR) at various thresholds. AUC values range from 0 to 1, where 1.0 indicates perfect classification, 0.5 indicates random guessing, and < 0.5 suggests poor performance (worse than random) [42]. Since it was proven that AUC is a better measure than accuracy in comparing learning algorithms [43], we used it to determine the best performing model.

Calibration is a degree of compliance between the probabilities predicted for each class and the accuracy of classifier on that prediction [44]. In our research, the Brier score was used as a measure of calibration. It should be noted that, unlike other metrics, a lower Brier score indicates better performance, that is, better model calibration [45].

### 2.5. Interpretative Framework

SHAPs represent one of the state-of-the-art ML interpretability models with a high extendibility. It is capable of calculating the contribution of each feature to the predicted outcome and visually represents it as the importance ranking [24, 46, 47]. SHAP can help us identify the output of the given classifier and understand the ML model decision-making process as valid and justified [20]. In this research, the models were represented using bar plots, which showed the degree of contribution, and violin plots, which visualized the overall correlation and directionality between features and the SHAP value [48].

LIMEs represent another interpretable model, which has shown itself useful in explaining individual samples or cases [24, 49]. This model is aimed at explaining the prediction-making process of any black-box model, by training a simpler interpretable model on a local subset of data around the example being explained [50]. The graph used in this analysis shows the overall predicted probability of a specific outcome on the left, decision-making process details in the middle, and the features' values and categories on the right.

### 2.6. Dense Artery, mRS, Age, Glucose, Onset-to-Treatment Time, and National Institutes of Health Stroke Scale (DRAGON) Score

Built models were compared mutually and with a previously established prognostic score—the DRAGON score. The DRAGON score, a 10-point measure scale, has proven itself reliable in supporting clinical decisions. Encompassing six baseline variables (hyperdense cerebral artery CT sign, prestroke mRS, age, baseline glucose level, onset-to-treatment time, and baseline NIHSS), the score showed high predictive power for both good (mRS score 0–2) and miserable outcomes (mRS score 5–6) [51, 52].

To generate the ROC curve, we used the training dataset to estimate the probabilities of unfavorable outcomes (mRS 3–6) associated with each specified DRAGON score *d*, denoted as *P*_unfavorable_(*d*):

(5) $$\begin{array}{c} P_{\text{unfavorable}}\left(d\right)=\frac{\text{number of patients with unfavorable outcomes }\left(d\right)}{\text{total number of patients in the training dataset }\left(d\right)} \end{array}$$

where *d* indicated the subset of patients with a DRAGON score of *d*, and *d* can be any of the 10 possible DRAGON score values.

These probabilities were then compared against the true mRS (score 3–6) labels for every instance in the test set. In accordance with the definition for calculating the ROC curve, based on the probabilities and true labels, for each threshold *t* from 0 to 1 applied on the probability, the TPR and FPR metrics were calculated as follows:

(6) $$\begin{array}{c} \text{True positive rate}=\frac{\text{true positives}}{\text{true positives}+\text{false negatives}} \end{array}$$

(7) $$\begin{array}{c} \text{False positive rate}=\frac{\text{false positives}}{\text{false positives}+\text{true negatives}} \end{array}$$

Finally, the ROC curve was plotted, with TPR on the *y*-axis and FPR on the *x*-axis.

## 3. Results

### 3.1. Sample Analysis

Our sample included 355 patients, with a median age of 67 years (IQR 60–74 years), 66% being male, and a majority (*n* = 196) having favorable 90-day outcomes. Patients with favorable outcomes were younger (65 years (IQR 57–70) vs. 71 years (IQR 63.5–77.0), *p* < 0.001), with lower values of baseline, 2-h, 24-h, and discharge NIHSS. They were hospitalized for a shorter time (12 days (IQR 7–15) vs. 15 days (IQR 10–22), *p* < 0.001), and their in-hospital stay was more commonly without complications (88% vs. 59%, *p* < 0.001). Described groups were compared, and the results of the statistical analysis are summarized in Table 3. The presented results are solely used to describe the sample and were not included in further analysis.

### 3.2. Classifiers

A complete table showing the best parameters for SVM, LR, and RF obtained from grid search is given in Table 4. In the case of SVM classifier, other than the linear SVM classifier, during the grid search process, the radial basis functions (rbfs) and sigmoid kernel were also considered. However, the results confirmed that the linear SVM was indeed the optimal choice for all the analyzed datasets.

The parameters [53] tuned via grid search for the SVM model included *C*, kernel, and gamma. The parameter *C* serves as a regularization parameter for controlling the penalty for misclassification. The kernel function transforms the input data into a higher-dimensional feature space, where the data becomes linearly separable. The gamma parameter, a coefficient of the kernel function, affects the decision boundary only if the chosen kernel function is “rbf,” “poly” (polynomial), or “sigmoid.” In the grid search, gamma was tuned alongside other parameters. However, since the optimal kernel identified for the model was “linear” in all cases, the gamma parameter had no impact on the model's performance.

For the LR models, the tuned parameters [54] were *C*, solver, and class weight. Similar to the SVM, parameter *C* controls the regularization strength. The solver specifies the algorithm used to solve the optimization problem, aiming at finding the best possible fit for the training data. Class weight is used to deal with imbalanced class proportions by assigning different weights to different classes. When class weight is set to “none,” all classes are treated equally, and each class is given the same importance when the SVM model is trained.

For the third classifier, RF, the tuned parameters [55] were as follows: number of estimators, max depth, min sample split, min sample leaf, max features, and criterion. As its name suggests, the number of estimators refers to the number of decision trees (estimators) in the RF. The remaining parameters apply to each individual decision tree. The max depth parameter helps prevent overfitting by limiting the number of nodes in each decision tree [56]. Setting the max depth parameter to “none” means that there is no limit on the depth of the individual decision trees in the forest. Min sample split specifies the minimum number of samples required to split an internal node during the tree-building process. Min sample leaf defines the minimum number of samples required to be at the terminal node (leaf node). Max features determine the maximum number of features to consider when searching for the best split. If max feature parameter is set to “sqrt,” it means that square root of the total number of features will be used as maximum. Finally, the criterion parameter defines the function used to measure the quality of each split.

### 3.3. Model Evaluation

The evaluation metrics of models derived in 10-fold CV performed on a training set are given in Table 5, and metrics derived on a test set are given in Table 6.

On the testing set, several measures were used to give a comprehensive understanding of how well the classifiers were performing. For all classifiers, performance generally improved from the baseline to the discharge model, except for the relationship between the baseline and the 2-h models, where the values were slightly smaller in the second time point. SVM appeared to be the best overall classifier, with the highest accuracy, precision, sensitivity, F1 score, and AUC in most time points. LR demonstrated strong sensitivity (0.879 in the discharge model), but its performance in accuracy and precision was generally surpassed by SVMs and RFs. RF performed best in terms of sensitivity in the 24-h and discharge models and achieved the best Brier score in the discharged model. However, it had lower metrics in the baseline and the 2-h models, particularly in sensitivity.

**Figure 3:**
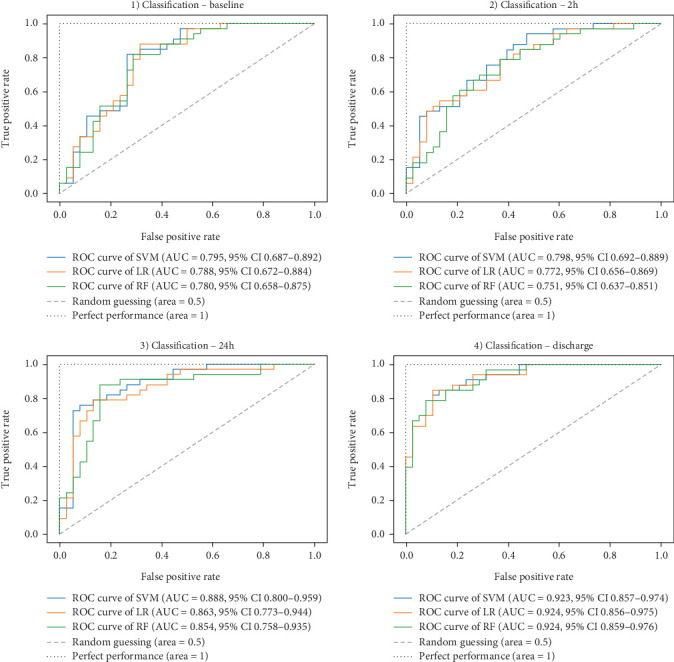
ROC curves of classifiers at different time points. The AUC values show that the best performing ones are as follows: SVM as the best baseline, 2-h, and 24-h model and RF as the best discharge model. They were further used for interpretable analysis. Random guessing is represented by the dashed line, with an AUC of 50% (0.5), and perfect performance with the dotted line (AUC of 100%). ROC: receiver operating characteristic curve, AUC: an area under the curve, CI: confidence interval, SVM: support vector machine, LR: logistic regression, RF: random forest.

**Figure 4:**
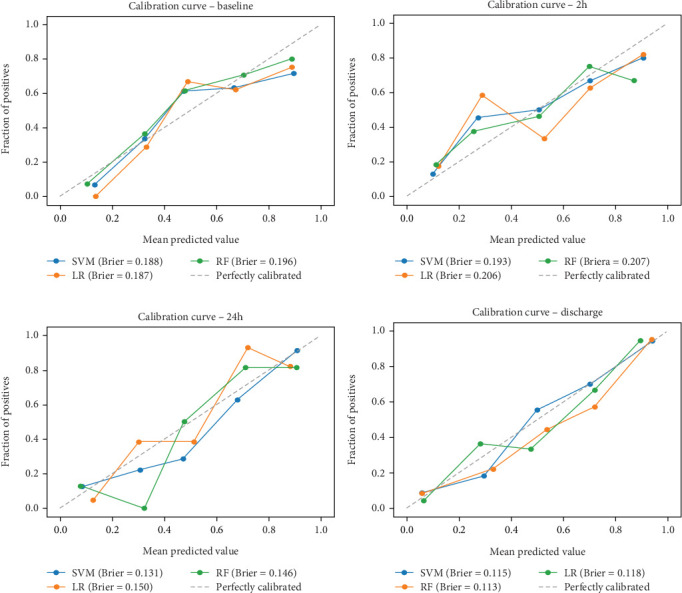
Models' reliability diagrams. Perfect calibration is represented by the dashed line. SVM, support vector machine; LR, logistic regression; RF, random forest.

Ultimately, AUC parameter was chosen for classifier comparison, and it was determined that the best classifier at each time point was as follows: SVM as the best baseline, 2-h, and 24-h model and RF as the best discharge model. The ROC curves calculated for the test phase are shown in Figure 3. The best models were further used for interpretable analysis. The models' reliability diagrams are shown in Figure 4, and confusion matrices are presented in Figure S1.

### 3.4. Interpretation Analysis—SHAP and LIME

**Figure 5:**
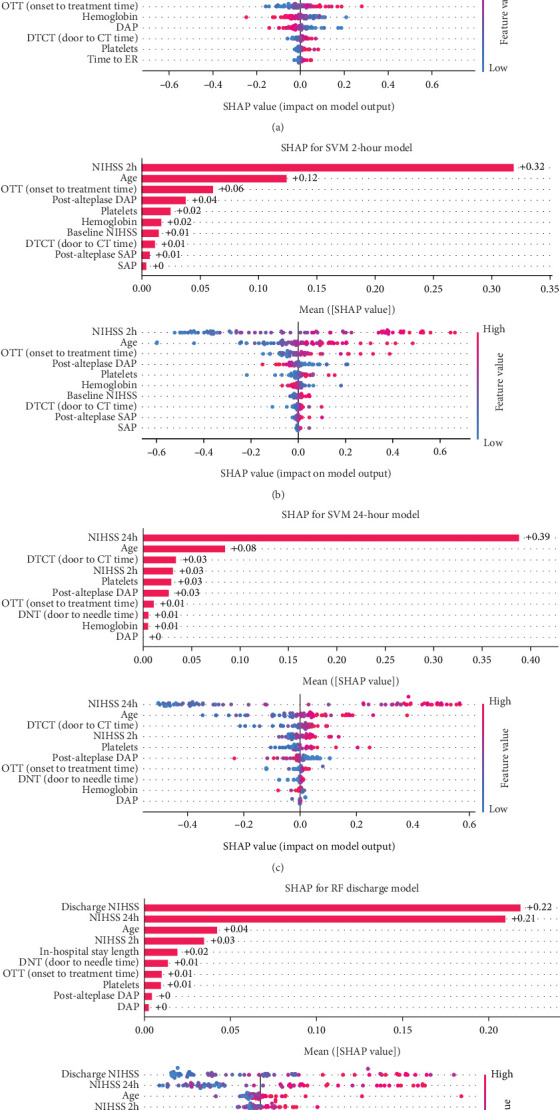
A feature-level interpretation using Shapley additive explanations (SHAPs). The influence of features on the unfavorable outcome has been presented through bar plots, showing the degree of contribution, and beeswarm plots, visualizing the overall correlation and directionality between features and the SHAP value. A correlation between the feature and the unfavorable outcome is indicated by the beeswarm plot, in which an increase in the intensity of the red color signifies a stronger correlation with the unfavorable outcome (mRS ≥ 3). The graphs were visualized for all the time points, including (a) baseline, (b) 2-h, (c) 24-h, and (d) and discharge models. NIHSS: the National Institutes of Health Stroke Scale, DAP: diastolic arterial pressure, ER: emergency room, SAP: systolic arterial pressure.

**Figure 6:**
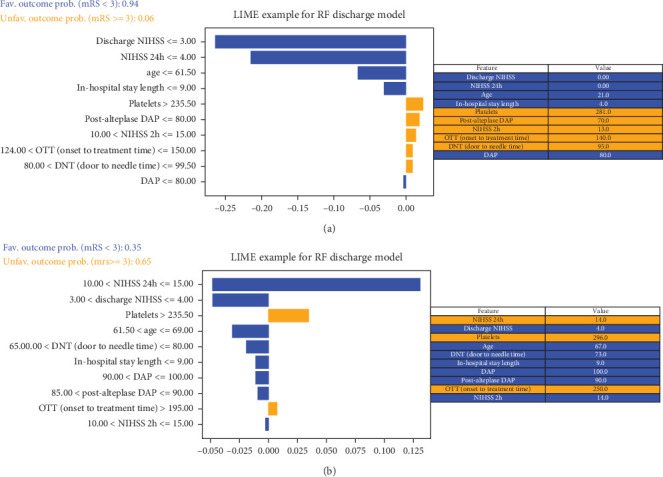
Local interpretable model–agnostic explanation (LIME) plot for individual case explanation on two random patients from the testing set of the RF discharge model. (a) A patient from a true negative group explained by the LIME algorithm and (b) a patient from a true negative group, explained by the LIME algorithm. NIHSS: the National Institutes of Health Stroke Scale, DAP: diastolic arterial pressure, ER: emergency room, SAP: systolic arterial pressure.

The decision-making process of the best-performing classifier, for each time point, was visualized using interpretable packages. Results of the SHAP analysis, as a feature-level interpretation method, are visualized in Figure 5, while the results of an individual-level interpretation model, or LIME, are shown in Figure 6.

#### 3.4.1. Baseline Model Interpretation

Based on the SHAP analysis performed on the baseline model (Figure 5a), baseline NIHSS value emerged as the most influential factor, followed by age, glycemia, and hemoglobin values. Based on the analysis of overall impact, higher value of baseline NIHSS, older age, higher value of glycemia, and lower hemoglobin values are important predictors of unfavorable 90-day outcomes.

#### 3.4.2. Two-Hour Model Interpretation

Analyzing the order of importance for the 2-h model, the 2-h value of NIHSS, age, onset-to-treatment time, and postalteplase diastolic pressure showed the highest impact. A favorable 90-day outcome was associated with lower values of the first three parameters and higher values of diastolic pressure (Figure 5b).

#### 3.4.3. Twenty-Four-Hour Model Interpretation

After 24 h, crucial parameters were a 24-h value of NIHSS, age, 2-h value of NIHSS, and platelet count. Like the previous models, higher values of NIHSS score and age were important predictors of unfavorable 90-day outcomes (Figure 5c).

#### 3.4.4. Discharge Model Interpretation

The discharge model has been shown as the most powerful in the prediction of 90-day outcomes. Features like 24-h and discharge values of NIHSS, age, and door-to-needle time resulted in a high predictive power of a model. Graphs showing the order of features' importance and overall impact on the prediction of the LR-based discharge model are shown in Figure 5d.

### 3.5. Comparison of Models

**Figure 7:**
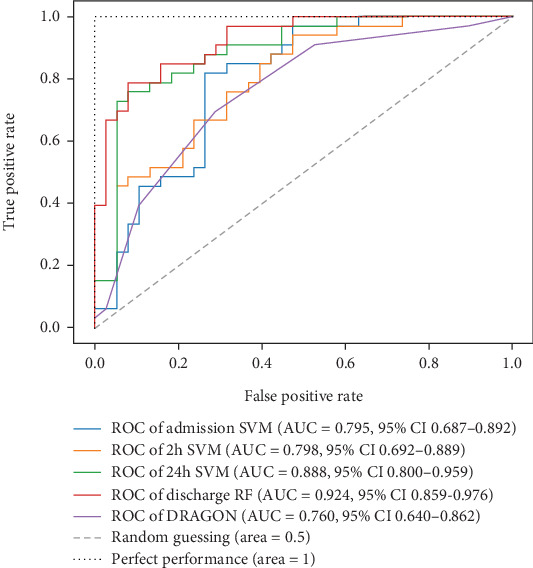
ROC curves comparing four built models and the model based on the DRAGON score. The area under the curve (AUC) values, along with their 95% confidence intervals, demonstrate the predictive performance of each model at four different time points. Random guessing is represented by the dashed line, with an AUC of 50% (0.5), and perfect performance with the dotted line (AUC of 100%). ROC: receiver operating characteristic curve, AUC: an area under the curve, CI: confidence interval, SVM: support vector machine, RF: random forest.

For model comparison for different time points, AUC scores were used as an evaluation metric. The model based on the DRAGON score showed an AUC value of 0.760 (95% confidence interval (CI), 0.640–0.862). When compared, trained models outperformed the model based on the DRAGON score, and the difference became even more pronounced when compared to the 24-h and discharge models (Figure 7).

## 4. Discussion

To the best of our knowledge, this is the first study that performed dynamic outcome prediction of alteplase-treated ischemic stroke patients using interpretable ML models. In this research, four models were built and internally evaluated, each with a different time point at which predictions were made. They showed moderate to high predictive power, and most of the newly constructed models outperformed the model based on the DRAGON score. Decision-making processes were visualized using interpretable packages, such as SHAPs and LIMEs; giving a better understanding of the matter; and providing a higher level of transparency.

### 4.1. Downsides of Preconstructed Predictive Models and Scores

In the context of precision medicine in stroke care, a more individualized approach is needed to provide a personalized outcome prediction [57]. Using predominantly admission data, the medical community has created multiple scores that forecast a patient's functional outcome. Some notable examples are Acute Stroke Registry and Analysis of Lausanne (ASTRAL), DRAGON, and Totaled Health Risks in Vascular Events (THRIVE). The resulting statistical models are considerably simplified to create integer-based scores since these scores are intended to be easily calculated by people using admission data. Consequently, the number of covariates is artificially decreased, and the weights of the models are discretized, which potentially worsens the models' performance [19]. Besides these, some other multivariable models have been developed and validated. In most of the models, routinely collected features, such as age, sex, stroke severity, and comorbidities (e.g., atrial fibrillation and diabetes mellitus), consistently appeared as predictor variables of stroke patients' functional outcomes. Even though methodological improvement and better model performances have been observed in previous years, if these models were externally evaluated, the discriminative power in terms of the 90-day favorable outcome (mRS ≤ 2) prediction was inconsistent (0.60 (95% CI, 0.57–0.64)–0.94 (95% CI 0.91–0.96)), raising concerns over reliability and usability of the models, as well as failure to assess clinical impact [10, 58]. It is also noteworthy that the majority of the models were developed in populations from developed countries [10], and that, due to possible racial and ethnic differences of the groups, various patients' backgrounds, disparities in the healthcare system, hospital type, and/or available acute stroke treatment, although valuable, predefined prognostic models may not be the best option in all cohorts of AIS patients [2].

### 4.2. Adaptability of ML Models to the Dynamic Clinical Environment

After experiencing a stroke, the patient's outcome depends on the various factors linked to different time frames of the recovery, and in a real clinical scenario, timing is another significant factor that influences treatment decisions [8, 59]. Therefore, prediction in only one time point may not be the most appropriate way to exploit the real power of the predictive models. In literature, the dynamic approach was more commonly used in patients treated with MT, as variables were divided into pre- and postinterventional datasets, observing higher predictive power when both types of features were included [14, 33]. In our study, we have focused on some of the most commonly used time points, as described in the previous study [19]. However, it is questionable whether these are the most appropriate time points since the real definition, timing, and duration of the critical period in stroke patients' recovery are still unknown [60]. This represents a novel opportunity for the use of dynamic models, as, potentially, they could not only help us in the identification of this period but also guide us through it, resulting in a higher degree of patient functionality. The most important potential benefit of dynamic prediction is a timely adjustment of the treatment, possibly improving patients' functional outcomes. One of ML's main advantages is its ability to automatically learn from and adapt to changing settings. It can change without requiring the system designer to anticipate and address every possible scenario, which is particularly useful given the difficulty of a consistently changing and dynamic clinical environment [61–63]. Therefore, future studies could include more time points, resulting in a more precise prediction system.

### 4.3. ML-Based Model Interpretability as the Missing Link for Clinical Application

The usage and application of ML models were enhanced by the improvements in computational power, data availability, and dimensionality, which led to an increased interest in these systems. The models, although optimized for task performance and factors like safety, fairness, reducing technical debt, and providing explanations, when used, carry a risk of new legal and ethical issues [64–66]. Due to a sharp increase in ML model usage, a critical moment for ML in medicine has been reached, and a need for developing a methodological standard has emerged [65]. In 2024, the updated version of Transparent Reporting of a multivariable prediction model for Individual Prognosis Or Diagnosis (TRIPOD) was published, additionally including models based on ML (TRIPOD + AI). It offers a comprehensive item checklist stating reporting recommendations aiming at promoting complete, accurate, and transparent reporting of research based on the previously described models [67]. Although extremely valuable, this guideline has not included the interpretation of models, which is one of the main conditions for their clinical usage. Interpretability, on its own, is a poorly defined concept, and there is little consensus on its usage in ML [64, 68, 69]. It is described as the ability to trace back how ML models generate their results, as well as explain and present them in understandable terms to a human [64, 70, 71]. Interpretation is described as relevant if it sheds light on a selected domain problem for a specific audience [68]. The interpretable models showed potential in overcoming the incompleteness of the results in terms of limited understanding of the problem, safety, and ethics, leading to a better scientific understanding and certainty when making decisions [64, 72]. For patient consent and well-informed treatment decisions, it is essential to adequately understand the input–output relationship of a model, and in this scenario, the model interpretability is one of the primary obstacles identified to the widespread implementation of these methodologies [65, 72]. Since the reasoning behind the model's behavior is crucial when making a decision, both for clinicians and patients, interpretability could ensure trust in the model, facilitating its clinical applicability [65, 66, 68, 69, 72, 73]. For this purpose, model-agnostic explanation methods such as SHAP and LIME have been developed, and they represent two commonly used techniques for model behavior analysis, as well as visualization of feature interactions and importance [65, 74–76]. Interpretable models demonstrate the surprisingly high utility of straightforward but precise models in practical applications, and they are essential for maintaining trust [73]. Based on everything said, we highly advocate for dynamic, interpretable ML model usage, as a multi–timepoint approach, followed by adequate explanations, could potentially increase the quality of stroke patients' care.

### 4.4. Similar Studies

Most of the studies mentioned in Table 1 used a single time point approach, which predominantly relied on admission features, restricting their capacity to adapt to the dynamic recovery process in stroke patients. Although this approach produced good predictive results, studies that included additional time points into their models, such as those investigating patients treated with MT [13, 33], achieved better AUC values compared to the baseline-only models from the same studies. This suggests that a multi–time point approach could provide a more comprehensive understanding of patients' recovery and could yield superior results. In addition, several studies done in both IVT- and MT-treated patients employed SHAP for interpretability [26, 31, 32]. While this method excels at feature-level interpretation and highlights overall importance in prediction, it does not provide patient-specific insights. For clinicians, understanding the reason why a specific patient is categorized into a particular outcome group still remains crucial for trust. As demonstrated in our study, LIME is capable of providing patient-specific interpretability, addressing the previously mentioned challenge. This capability highlights its potential and emphasizes the need for its broader adoption in predictive modeling.

### 4.5. Study Limitations

This study has several limitations. First, as the main goal of this study was to test a concept, the number of participants is relatively small. Therefore, a larger sample size is needed to validate these models for use in a real clinical environment. Second, models can predict the outcomes of only alteplase-treated patients, meaning that they cannot be used in patients treated with other modalities, such as tenecteplase or interventional approaches. Third, future models could implement more advanced techniques, such as DL, to exploit raw neuroradiological data and by that increase the predictive power of the models. While designing the study, we considered using DL models. However, DL is particularly advantageous for large, high-dimensional datasets, such as those involving images, text, or audio [77]. In contrast, for smaller, low-dimensional datasets, shallow ML models have been shown to outperform DL models and are generally easier to interpret [71, 78]. Given that our dataset is relatively small and does not include radiological images, ML models were deemed more suitable for this study. Fourth, it is worth mentioning that a central question during a supervised ML model build-up process concerns the accuracy of the resulting model and that a key problem is overfitting. Ideally, to prevent this problem, the model would be evaluated using new data originating from the same population. However, in practice, this is usually not feasible, and therefore, resampling methods, such as CV, play an important role in overfitting prevention [79]. Even though, in this study, the CV method was used in the analysis, more data should be collected, and the predictive model certainly needs to be evaluated in a real clinical environment.

### 4.6. Contribution and Future Directions

The main contribution of our research and proposed method lies in the comprehensive multi–time point utilization of routinely documented clinical data to gain valuable insights into the prognostics of AIS patients. The nature of the data used and the transparent understanding of the decision-making process make the models potentially implementable in everyday practice, provided the system is integrated with the hospital information system. However, we must acknowledge that this is a proof-of-concept study, and further investigations are needed to validate and build upon these findings. In the future, more complex models could be developed to directly incorporate CT/MRI images into the classifier, which, based on previous research, could further enhance outcome prediction.

## 5. Conclusions

To the best of our knowledge, this study is the first to employ interpretable ML models for dynamic prediction of 90-day functional outcomes in alteplase-treated ischemic stroke patients. Four models were built and evaluated, each with a different time point at which predictions were made. They showed moderate to high predictive power, and almost every newly constructed model outperformed the model based on the DRAGON score. In all constructed models, age, onset-to-treatment time, and platelet count were recognized as the important predictors, followed by clinical parameters measured at different time points, such as the NIHSS, systolic, and diastolic blood pressure values. Decision-making processes were visualized using interpretable packages SHAP and LIME, giving a better understanding of the matter, and providing a higher level of transparency. The dynamic approach, coupled with interpretable models, can aid in providing insights into the potential factors that could be modified and thus contribute to a better outcome in this patient group.

## Figures and Tables

**Table 1:** Comparison of selected studies using machine learning– and deep learning–based models for stroke outcome prediction.

**No.**	**Study**	**Study type**	**Goal**	**Dataset description**	**Models used**	**Features**	**AUC**	**Key findings**	**Interpretability: Most significant factors**	**Multi–time point approach**
1.	Abujaber et al., 2023 [26]	Retrospective study	Predicting favorable 3-month functional outcome (mRS 0–2)	723 AIS patients treated by IVT	XGB, RF, SVM, LR, CART	Demographic and clinical data	XGB: 0.756; RF: 0.758; SVM: 0.763; LR: 0.719; CART: 0.623	ML models effectively predict 3-month functional outcomes	Feature-level interpretation (SHAP): Baseline NIHSS, prestroke mRS	Baseline factors and hospitalization data (hospital-acquired pneumonia and urinary tract infection)
2.	Park et al., 2021 [27]	Retrospective study with a prospective cohort database	Predicting favorable 3-month functional outcome (mRS 0–1)	1066 AIS patients	Regularized LR, SVM, RF, KNN, XGBoost	Demographics, stroke-related factors, lab findings, comorbidities	LR: 0.86 (IQR 0.82–0.90), SVM: 0.85 (IQR 0.81–0.89), RF: 0.82 (IQR 0.77–0.87), KNN: 0.82 (IQR 0.77–0.87), XGB: 0.81 (IQR 0.76–0.86)	ML models effectively predict 3-month functional outcomes	Feature-level interpretation: NIHSS and age	No
3.	Hatami et al., 2023 [28]	Prospective monocentric observational cohort	Predicting favorable 3-month functional outcome (mRS 0–2)	119 anterior circulation LVO-AIS patients treated by MT	Autoencoder-LSTM model	MRI data	AUC: 0.71 ± 0.03	The proposed AE2-LSTM model outperforms existing models in stroke outcome prediction	No	Time series data
4.	Tsai et al., 2024 [29]	Retrospective study	Predicting the 3-month functional outcome (mRS > 2)	3297 AIS patients	A deep fusion learning network	Diffusion-weighted MRI and clinical data	AUC: 0.87	Fusion model outperforms existing models; MRI can replace NIHSS for prediction	Class activation mapping (CAM)	No
5.	Heo et al., 2019 [30]	Retrospective study using a prospective cohort	Predicting favorable 3-month functional outcome (mRS 0–2)	2604 AIS patients	DNN, RF, LR	Demographics, clinical variables	DNN: 0.888 (95% CI, 0.873–0.903), RF: 0.857 (95% CI, 0.840–0.874), LR: 0.849 (95% CI, 0.831–0.867)	DNN significantly outperformed ASTRAL and other models for long-term outcome prediction; ML showed potential to enhance treatment decisions in AIS	No	No
6.	Jabal et al., 2022 [31]	Retrospective study	Predicting favorable 3-month functional outcome (mRS 0–2)	293 anterior circulation LVO-AIS patients treated by MT	KNN, RF, GB, XGB	Clinical and imaging features	KNN: 0.76; RF: 0.73; GB: 0.75; XGB: 0.80	SHAP identified key predictors like age, NIHSS score, and CTA-clot burden score	Feature-level interpretation (SHAP): Age and baseline NIHSS	No
7.	Yao et al., 2022 [32]	Retrospective study	Predicting favorable 3-month functional outcome (mRS 0–2)	217 anterior circulation LVO-AIS patients treated by MT	Base models: AdaBoost, LightGBM, XGBoost, random forest, gradient boosting, extra trees, CatBoost Final model: PFCML-MT	Clinical and imaging features	Base models: AUC 0.83–0.90; final model: AUC 0.84–0.87	Developed interpretable models	Feature-level interpretation (SHAP): Baseline serum glucose and baseline NIHSS	Three time points: Preoperative, intraoperative, and within 1 day postoperatively
8.	Petrović et al., 2024 [33]	Retrospective study	In-hospital death	602 anterior circulation LVO-AIS patients treated by MT	Preprocedural (pre-MT) and postprocedural (post-MT) models: LR, RF, GB, XGB	Clinical, laboratory, and imaging features	Pre-MT: AUC 0.792; post-MT: AUC 0.837	Demonstrated effectiveness of interpretable ML for predicting in-hospital mortality after MT	Feature-level interpretation (SHAP): Baseline NIHSS, age, and peripheral arterial disease; individual-level interpretation (LIME)	Two time points: Preoperatively and postoperatively
9.	Sommer et al., 2024 [34]	Retrospective study	Predicting favorable 3-month functional outcome (mRS 0–2)	591 anterior circulation LVO-AIS patients treated by MT	Ensemble model 1. CTA data 2. CTA data + treatment data 3. CTA data + treatment data + clinical data	Radiological and clinical features	CTA: 0.70 (IQR 0.59–0.81), CTA + treatment: 0.79 (IQR 0.70–0.89), CTA + treatment + clinical: 0.86 (IQR 0.79–0.94)	Demonstrated feasibility for automated prognostication, aiding telehealth and scenarios with limited neurological examination	Utilized M3d-CAM to improve interpretability by highlighting key regions in head CTA scans	No
10.	Hu et al., 2022 [13]	Retrospective study	Predicting an unfavorable outcome at the 3-month mark (mRS 3–6)	239 LVO-AIS patients treated by MT	XGB	Clinical, laboratory, and radiological data	Admission: 0.835; 24 h: 0.917; 3 days: 0.937; discharge: 0.987	The first dynamic pre- and postoperative predictive model for AIS patients undergoing MT; improving accuracy over previous models	No	Admission, 24-h, 3-day, and discharge models

Abbreviations: AdaBoost, adaptive boosting; AE2-LSTM, two-level autoencoders followed by a long short-term memory; AIS, acute ischemic stroke; AUC, area under the curve; CAM, class activation mapping; CART, classification and regression trees; CTA, computed tomography angiography; DNN, deep neural network; GB, gradient boosting; IQR, interquartile range; IVT, intravenous thrombolysis; KNN, *k*-nearest neighbors; LightGBM, light gradient boosting machine; LIME, local interpretable model–agnostic explanations; LR, logistic regression; LVO, large vessel occlusion; ML, machine learning; MRI, magnetic resonance imaging; mRS, modified Rankin scale; MT, mechanical thrombectomy; NIHSS, National Institutes of Health Stroke Scale; RF, random forest; SHAP, Shapley additive explanation; SVM, support vector machine; XGB, extreme gradient boosting.

**Table 2:** Time point–based feature division with feature types.

**Time point**	**Features**	**Feature type**
Baseline features	Age, sex (cat.), body weight	Patient data
	Baseline NIHSS, baseline SBP, baseline DBP, baseline mean BP	Clinical data
	Hemoglobin, glycemia, platelets, aPTT, PT-INR	Laboratory data
	Hyperdense CT sign (cat.), leukoaraiosis (cat.), ASPECTS	Neuroradiological assessment
	Prior usage of drugs (acetylsalicylic acid (cat.), clopidogrel (cat.), oral anticoagulant treatment (cat.), statins (cat.), antihypertensive drugs (cat.)), Hypertension (cat.), diabetes mellitus (cat.), tobacco smoking (cat.), hyperlipoproteinemia (cat.), atrial fibrillation (cat.), cardiomyopathy (cat.), alcohol consumption (cat.)	Vascular risk factors
	Time to ER, OT time, DN time, door to CT time, dose of alteplase, BP reduction	Treatment data
	OCSP type of stroke (cat.), TOAST classification (cat.)	Stroke data

2-h features	NIHSS 2 h	Outcomes and discharge data
	Postalteplase SBP, postalteplase DBP, postalteplase mean AP	Treatment data

24-h features	NIHSS 24 h, early neurological improvement 24 h (cat.)	Outcomes and discharge data
	Hemorrhagic transformation (cat.), symptomatic intracerebral hemorrhage (cat.)	Treatment data

Discharge features	Discharge NIHSS, discharge treatment (cat.), facility of discharge (cat.)	Outcomes and discharge data
	Postalteplase cholesterol value	Treatment data
	In-hospital stay length, complications (cat.)	Hospitalization data

*Note*: For clarity, categorical variables were denoted with “cat.” (cat.: categorical feature).

Abbreviations: AP: arterial pressure, ASCPETS: Alberta Stroke Program Early CT Score, BP: blood pressure, DBP: diastolic blood pressure, DN: door to needle, ER: emergency room, NIHSS: National Institutes of Health Stroke Scale, OCSP: Oxfordshire Community Stroke Project, OT: onset to treatment, SBP: systolic blood pressure, TOAST: Trial of Org 10172 in Acute Stroke Treatment.

**Table 3:** Statistical analysis of the sample.

**Variable**	**All patients** **(** **n** = 355**)**	**Patients with favorable outcome (** **n** = 196**)**	**Patients with unfavorable outcome (** **n** = 159**)**	**p** ** value**
Patient data				
Age (years), median (IQR)	67 (60–74)	65 (57–70)	71 (63–77)	< 0.001
Sex, *n* (%)				0.354
Male	227 (64%)	130 (66%)	97 (61%)	
Female	128 (36%)	66 (34%)	62 (39%)	
Bodyweight (kg), median (IQR)	82 (74–90)	83 (745–90)	80 (73–90)	0.421
Clinical Data				
Baseline NIHSS, median (IQR)	13 (9–17)	10 (7–15)	16 (12–18)	< 0.001
Baseline systolic blood pressure (mmHg), median (IQR)	155 (140–170)	150 (140–165)	160 (145–170)	0.011
Baseline diastolic blood pressure (mmHg), median (IQR)	90 (80–100)	90 (80–96)	90 (79–100)	0.971
Baseline mean blood pressure (mmHg), median (IQR)	110 (100–120)	110 (100–120)	112 (102–122)	0.189
Laboratory data				
Hemoglobin (g/L), median (IQR)	142 (131–151)	144 (133–152)	140 (128–149)	0.057
Glycemia (mmol/L), median (IQR)	7.00 (6.20–8.90)	6.75 (6.00–8.10)	7.50 (6.40–9.65)	< 0.001
Platelets (× 10^9^/L), median (IQR)	219 (182–260)	217 (182–254)	221 (181–273)	0.101
aPTT (seconds), median (IQR)	23.40 (0.98–26.60)	23.4 (0.97–26.52)	23.4 (0.98–26.60)	0.826
PT-INR, median (IQR)	1.03 (0.99–1.10)	1.03 (0.98–1.08)	1.04 (0.99–1.12)	0.247
Neuroradiological assessment				
Hyperdense CT sign, *n* (%)	113 (32%)	49 (25%)	64 (40%)	0.003
Leukoaraiosis, *n* (%)	63 (18%)	25 (13%)	38 (24%)	0.010
ASPECTS, median (IQR)	10 (9–10)	10.0 (9.0–10.0)	10.0 (8.0–10.0)	0.001
Vascular risk factors				
Acetylsalicylic acid, *n* (%)	108 (30%)	68 (35%)	40 (25%)	0.068
Clopidogrel, *n* (%)	11 (3%)	5 (3%)	6 (4%)	0.724
Oral anticoagulant treatment, *n* (%)	5 (1%)	2 (1%)	3 (2%)	0.813
Statins, *n* (%)	40 (11%)	22 (11%)	18 (11%)	1.000
Antihypertensive drugs, *n* (%)	235 (66%)	119 (61%)	116 (73%)	0.021
Hypertension, *n* (%)	311 (88%)	162 (83%)	149 (94%)	0.003
Diabetes mellitus, *n* (%)	67 (19%)	26 (13%)	41 (26%)	0.004
Tobacco smoking, *n* (%)	108 (30%)	64 (33%)	44 (28%)	0.369
Hyperlipoproteinemia (HLP), *n* (%)	165 (46%)	109 (56%)	56 (35%)	< 0.001
Without HLP	190 (54%)	87 (44%)	103 (65%)	< 0.001
HLP Type IIa	81 (23%)	56 (29%)	25 (16%)	0.006
HLP Type IIb	54 (15%)	30 (15%)	24 (15%)	1.000
HLP Type IV	30 (8%)	23 (12%)	7 (4%)	0.023
Atrial fibrillation, *n* (%)	119 (34%)	51 (26%)	68 (43%)	0.001
Cardiomyopathy, *n* (%)	57 (16%)	32 (16%)	25 (16%)	0.993
Alcohol consumption, *n* (%)	8 (2%)	5 (3%)	3 (2%)	0.952
Treatment data				
Time to ER (minutes), median (IQR)	72 (50–113)	71 (48–114)	75 (52–113)	0.796
Onset to treatment time (minutes), median (IQR)	155 (122–200)	153 (125–201)	160 (121–199)	0.668
Door to needle time (minutes), median (IQR)	78 (61–97)	76 (61–95)	80 (61–100)	0.496
Door to CT time (minutes), median (IQR)	45 (30–60)	43 (30–58)	46 (30–64)	0.169
Dose of alteplase (mg), median (IQR)	73.8 (67–81)	75 (67–81)	72 (66–81)	0.327
Blood pressure reduction, *n* (%)	74 (21%)	34 (17%)	40 (25%)	0.095
Postalteplase systolic blood pressure (mmHg), median (IQR)	150 (135–160)	146 (135–160)	150 (140–165)	0.033
Postalteplase diastolic blood pressure (mmHg), median (IQR)	80 (75–90)	85 (75 - 90)	80 (74–90)	0.327
Postalteplase mean blood pressure (mmHg), median (IQR)	107 (96–113)	107 (95–113)	105 (97–115)	0.718
Postalteplase cholesterol value (mmol/L), median (IQR)	5.15 (4.61–5.92)	5.21 (4.72–6.02)	5.15 (4.36–5.62)	0.020
Hemorrhagic transformation, *n* (%)	64 (18%)	23 (12%)	41 (26%)	0.001
Symptomatic intracerebral hemorrhage, *n* (%)	*n* (3%)	0 (0%)	9 (6%)	0.002
Stroke data				
OCSP type of stroke, *n* (%)				
Total anterior circulation infarction (TACI)	97 (27%)	25 (13%)	72 (45%)	< 0.001
Partial anterior circulation infarction (PACI)	155 (44%)	97 (49%)	58 (36%)	0.019
Lacunar anterior circulation infarction (LACI)	51 (14%)	43 (22%)	8 (5%)	< 0.001
Posterior circulation infarction (POCI)	52 (15%)	31 (16%)	21 (13%)	0.589
TOAST classification, *n* (%)				
Cardioembolic (CE)	110 (31%)	45 (23%)	65 (41%)	< 0.001
Large artery atherosclerosis (LAA)	95 (27%)	45 (23%)	50 (31%)	0.094
Small vessel disease (SVD)	51 (14%)	40 (20%)	11 (7%)	0.001
Undetermined/other	99 (28%)	66 (34%)	33 (21%)	0.010
Side of visualized ischemic lesion, *n* (%)				
None	33 (9%)	29 (15%)	4 (3%)	< 0.001
Left	172 (48%)	80 (41%)	92 (58%)	0.002
Right	145 (41%)	84 (43%)	61 (38%)	0.455
Both	5 (1%)	3 (2%)	2 (1%)	1.000
Hospitalization data				
In-hospital stay length (days), median (IQR)	13 (8–18)	12 (7–15)	15 (10–22)	< 0.001
Complications, *n* (%)				
Pneumonia	30 (8%)	9 (5%)	21 (13%)	0.007
Urinary tract infection	36 (10%)	14 (7%)	22 (14%)	0.057
Deep vein thrombosis	3 (1%)	1 (1%)	2 (1%)	0.855
Cardiac decompensation	7 (2%)	1 (1%)	6 (4%)	0.069
Decubitus	3 (1%)	0 (0%)	3 (2%)	0.178
No complications	266 (75%)	172 (88%)	94 (59%)	< 0.001
In-hospital death, *n* (%)	34 (10%)	0 (0%)	34 (21%)	< 0.001
Outcomes and discharge data				
NIHSS 2 h, median (IQR)	10 (6–15)	8 (3–10)	15 (10–17)	< 0.001
NIHSS 24 h, median (IQR)	8 (3–15)	4 (2–6)	15 (11–18)	< 0.001
Early neurological improvement 24 h, *n* (%)	129 (36%)	118 (60%)	11 (7%)	< 0.001
Discharge NIHSS, median (IQR)	4 (2–9)	2 (1–4)	10 (4–14)	< 0.001
Discharge treatment, *n* (%)				
Antiplatelet drugs	240 (68%)	149 (76%)	91 (57%)	< 0.001
Double antiplatelet therapy	9 (3%)	6 (3%)	3 (2%)	0.718
Oral anticoagulant treatment	50 (14%)	34 (17%)	16 (10%)	0.071
Low molecular weight heparin	20 (6%)	6 (3%)	14 (9%)	0.036
No secondary prevention	36 (10%)	1 (1%)	35 (22%)	< 0.001
Facility of discharge, *n* (%)				
Home	189 (53%)	133 (68%)	56 (35%)	< 0.001
Rehabilitation centre	150 (42%)	61 (31%)	89 (56%)	< 0.001
Other healthcare provider	4 (1%)	0 (0%)	4 (3%)	0.084
Other	12 (3%)	2 (1%)	10 (6%)	0.015

Abbreviations: aPTT, activated partial thromboplastin time; ASPECTS, the Alberta Stroke Program Early CT Score; CT, computed tomography; ER, emergency room; IQR, interquartile range; NIHSS, the National Institutes of Health Stroke Scale; OCSP, the Oxfordshire Community Stroke Project; PT-INR, prothrombin time–international normalized ratio.

**Table 4:** Grid search output for the used classifiers: support vector machine (SVM), logistic regression (LR), and random forest (RF). The third column contains the best values for each of the four datasets that were analyzed—(I) baseline data, (II) 2-h data, (III) 24-h data, and (IV) discharge data—thus they are given in vector form.

**Classifier**	**Parameter**	**Values**
SVM	Kernel	[Linear, linear, linear, linear]
	*C* (regularization)	[10, 0.1, 0.1, 0.1]
	Gamma	[1, 100, 1, 1]

LR	*C* (inverse regularization)	[0.01, 0.1, 0.001, 100]
	Solver	[lbfgs, liblinear, liblinear, liblinear]
	Class weight	[Balanced, none, none, balanced]

RF	Number of estimators	[100, 200, 100, 200]
	Max depth	[None, 10, none, 10]
	Min sample split	[5, 10]
	Min sample leaf	[1, 2, 4]
	Max features	[sqrt, sqrt, sqrt, sqrt]
	Criterion	[Entropy, gini, entropy, gini]

**Table 5:** Evaluation metrics of classifiers obtained on the training set.

	**Baseline model**	**2-h model**	**24-h model**	**Discharge model**	**Classifier**
Accuracy (mean ± std)	0.740 ± 0.080	0.781 ± 0.043	0.824 ± 0.043	0.870 ± 0.056	SVM
	0.743 ± 0.073	0.799 ± 0.042	0.849 ± 0.039	0.877 ± 0.049	LR
	0.732 ± 0.060	0.813 ± 0.048	0.859 ± 0.032	0.866 ± 0.041	RF

*Note:* The largest mean value in each column is bolded.

Abbreviations: LR: logistic regression, RF: random forest, SVM: support vector machine.

**Table 6:** Evaluation metrics of classifiers obtained on the test set.

**Metric**	**Baseline model**	**2-h model**	**24-h model**	**Discharge model**	**Classifier**
Accuracy	**0.746**	0.690	0.817	**0.859**	SVM
	0.704	0.662	0.817	0.831	LR
	0.662	**0.704**	**0.859**	0.831	RF

Precision	**0.714**	0.657	0.812	**0.848**	SVM
	0.676	0.636	0.812	0.784	LR
	0.667	**0.676**	**0.829**	0.818	RF

Sensitivity	**0.758**	**0.697**	0.788	0.848	SVM
	0.697	0.636	0.788	**0.879**	LR
	0.545	**0.697**	**0.879**	0.818	RF

F1 score	**0.735**	0.676	0.8	**0.848**	SVM
	0.687	0.636	0.8	0.829	LR
	0.6	**0.687**	**0.853**	0.818	RF

AUC (95% CI)	**0.795 (0.687–0.892)**	**0.798 (0.692–0.889)**	**0.888 (0.800–0.959)**	0.923 (0.857–0.974)	SVM
	0.788 (0.672–0.884)	0.772 (0.656–0.869)	0.863 (0.773–0.944)	0.924 (0.856–0.975)	LR
	0.780 (0.658–0.875)	0.751 (0.637–0.851)	0.854 (0.758–0.935)	**0.924 (0.859–0.976)**	RF

Brier score	0.188	**0.193**	**0.131**	0.115	SVM
	**0.187**	0.206	0.15	0.118	LR
	0.196	0.207	0.146	**0.113**	RF

*Note:* The largest value of each metric in each column is bolded, except for the Brier score, as lower value indicated better calibration.

Abbreviations: AUC: area under the curve, CI: confidence interval, LR: logistic regression, RF: random forest, SVM: support vector machine.

## Data Availability

The data that support the findings of this study are available on request from the corresponding author. The data are not publicly available due to privacy or ethical restrictions.

## Supporting Information

Supporting Information Additional supporting information can be found online in the Supporting Information section. In the Supporting Information, Table S1 contains the descriptions of the features used during the analysis and Figure S1 visualizes the confusion matrices of the classifiers.
