# Supplementary material for: Dynamic, Interpretable, Machine Learning–Based Outcome Prediction as a New Emerging Opportunity in Acute Ischemic Stroke Patient Care: A Proof-of-Concept Study
Source: Stroke Res Treat. 2025 Mar 25;2025:3561616. doi: 10.1155/srat/3561616 (PMC11961286; doi:10.1155/srat/3561616)
Supplement: Supporting Information — Additional supporting information can be found online in the Supporting Information section. In the Supporting Information, Table S1 contains the descriptions of the features used during the analysis and Figure S1 visualizes the confusion matrices of the classifiers. [file 3561616.f1.docx]

**Supplementary material**

*Supplementary table 1*. Features description.

| **Feature** | **Type** | **Description** |
| --- | --- | --- |
| Age | Continuous | This parameter referred to patients' age at the time of stroke onset. |
| Sex | Categorical | Two categories: male and female |
| Bodyweight | Continuous | This parameter referred to patients’ weight based on patients’ statements. |
| Baseline NIHSS | Continuous | This parameter refers to the initial value of NIHSS. |
| 2-hour NIHSS | Continuous | This parameter refers to the value of NIHSS 2-hour after the beginning of thrombolysis. |
| 24-hour NIHSS | Continuous | This parameter refers to the value of NIHSS 24 hours after stroke onset. |
| Discharge NIHSS | Continuous | This parameter refers to the discharge value of NIHSS. |
| Baseline systolic blood pressure | Continuous | This parameter refers to the initial value of systolic blood pressure, and it was expressed in millimeters of mercury column (mmHg). |
| Baseline diastolic blood pressure | Continuous | This parameter refers to the initial value of diastolic blood pressure, and it was expressed in millimeters of mercury column (mmHg). |
| Baseline mean blood pressure | Continuous | This parameter refers to the initial value of mean blood pressure. It was calculated using a formula ((systolic blood pressure + 2xdiastolic blood pressure)/3), and it was expressed in millimeters of mercury column (mmHg). |
| Hemoglobin | Continuous | This parameter refers to the baseline value of hemoglobin, expressed in grams per liter (g/L). |
| Glycemia | Continuous | This parameter refers to the baseline value of glycemia, expressed in millimoles per liter (mmol/L). |
| Platelets | Continuous | This parameter refers to the baseline value of platelets, expressed in number of platelets x 10^9^ per liter. |
| aPTT | Continuous | This parameter refers to the baseline value of activated partial thromboplastin time (aPTT), expressed in seconds. |
| PT-INR | Continuous | This parameter refers to the baseline value of prothrombin time-international normalized ratio (PT-INR), expressed as an absolute number. |
| Hyperdense CT sign | Categorical | Based on the presence of hyperdense sign during the initial CT scan. Categories were: present or absent. |
| Leukoaraiosis | Categorical | Based on the presence of leukoaraiosis during the initial CT scan. Categories were: present or absent. |
| ASPECTS | Continuous | Based on the ASPECT score during the initial CT scan. |
| Acetylsalicylic acid | Categorical | Based on the previous usage of this medication. Categories were yes or no. |
| Clopidogrel | Categorical | Based on the previous usage of this medication. Categories were yes or no |
| Oral anticoagulant treatment | Categorical | Based on the previous usage of this medication. Categories were yes or no |
| Statins | Categorical | Based on the previous usage of this medication. Categories were yes or no |
| Antihypertensive drugs | Categorical | Based on the previous usage of this medication. Categories were yes or no |
| Hypertension | Categorical | Based on the presence of this risk factor in patients’ medical history. Categories were present or absent. |
| Diabetes Mellitus | Categorical | Based on the presence of this risk factor in patients’ medical history. Categories were: present or absent. |
| Tobacco smoking | Categorical | Based on the patient’s statement. Categories were smoker or non-smoker. |
| Hyperlipoproteinemia | Categorical | Based on the presence of this risk factor in patients’ medical history. Categories were present or absent. |
| Type of hyperlipoproteinemia | Categorical | Patients were divided into four categories: without, type IIa, type IIb, and type IV. |
| Atrial fibrillation | Categorical | Based on the presence of this risk factor in patients’ medical history, or during the admission. Categories were present or absent. |
| Cardiomyopathy | Categorical | Based on the presence of this risk factor in patients’ medical history. Categories were present or absent. |
| Alcohol consumption | Categorical | Based on the patient’s statement. Categories were present or absent. |
| Time to ER | Continuous | Times in minutes passed from the witnessed or self-reported onset of neurological symptoms to the moment when the patient arrived to the emergency room (ER). |
| Onset to treatment time | Continuous | Times in minutes passed from the witnessed or self-reported onset of neurological symptoms to the moment when the intravenous thrombolysis was initiated. |
| Door-to-needle time | Continuous | Times in minutes passed from the entrance to the ER to the moment when the intravenous thrombolysis was initiated. |
| Door to CT time | Continuous | Times in minutes passed from the entrance to the ER to performance of brain CT scan. |
| Dose of alteplase | Continuous | Dose of alteplase was expressed in mg. The dosing regimen implied 0.9mg/kg of body weight, with a maximum of 90mg.^32^ |
| Blood pressure reduction | Categorical | Based on the need of reducing patients blood pressure, two categories were formed: patients with high blood pressure that needed reduction prior to the alteplase treatment, and patients that didn’t need reduction. |
| Post-alteplase systolic blood pressure | Continuous | This parameter refers to the post-alteplase value of systolic blood pressure, which was measured in a time frame of 2 hours after the treatment initiation. It was expressed in millimeters of mercury column (mmHg). |
| Post-alteplase diastolic blood pressure | Continuous | This parameter refers to the post-alteplase value of diastolic blood pressure, which was measured in a time frame of 2 hours after the treatment initiation. It was expressed in millimeters of mercury column (mmHg). |
| Post-alteplase mean blood pressure | Continuous | This parameter refers to the post-alteplase value of mean blood pressure, which was calculated by a previously mentioned formula. It was expressed in millimeters of mercury column (mmHg). |
| Post-alteplase Cholesterol value | Continuous | This parameter refers to the value of total cholesterol, measured during the in-hospital stay. It was expressed in millimoles per liter (mmol/L). |
| Hemorrhagic transformation | Categorical | This parameter was based on the presence of absence of hemorrhagic transformation during a control CT scan, 24 hours after the treatment. Categories were present or absent. |
| Symptomatic Intracerebral Hemorrhage | Categorical | This parameter was defined as any intracerebral bleeding with neurological deterioration, including increase of ≥4 NIHSS points, or death related to the bleeding itself. Categories were present or absent. |
| OCSP Type of Stroke | Categorical | Categories were based on the Oxfordshire Community Stroke Project (OCSP) classification: (I) total anterior circulation infarct (TACI), (II) partial anterior circulation infarct (PACI), (III) lacunar infarct (LACI), and (IV) posterior circulation infarct (POCI).^33^ |
| TOAST classification | Categorical | Based on the Trial of ORG 10172 in Acute Stroke Treatment, 4 categories were made: (I) cardioembolism (CE), (II) large-artery atherosclerosis (LAA), (III) small-vessel disease (SVD), and (IV) Undetermined or other caue.^33^ |
| Side of Visualized Ischemic Lesion | Categorical | Based on the presence and side of ischemic changes during the initial CT scan, categories were absent, left side, right side, and both sides. |
| In-Hospital Stay Length | Continuous | This parameter included days spent |
| Complications | Categorical | Without, Pneumonia, UTI, Deep vein thrombosis, cardiac decompensation, decubitus |
| In-hospital death | Categorical | This parameter included two categories: patients that died during the in-hospital stay, and patients that did not die. |
| Early Neurological Improvement 24h | Categorical | In our study, early neurological improvement (ENI) was defined as a reduction of the baseline NIHSS score for ≥40%. |
| Discharge treatment | Categorical | Based on the prescribed treatment, 5 categories were made: (I) No secondary prevention, (II) Antiplatelet drugs, (III) Double antiplatelet drugs, (IV) Oral anticoagulant treatment, and (V) Low-molecular-weight heparin. |
| Facility | Categorical | Based on the facility to which the patient has been discharged, 4 categories were made: (I) Home, (II) Rehabilitation center, (III) Other healthcare provider (including hospitals, and secondary level healthcare providers), and (IV) Other, if a patient was discharged to some other facility that was not listed previously. |

NIHSS, National Institutes of Health Stroke Scale; ASCPETS, Alberta Stroke Program Early CT Score; ER, Emergency Room; OCSP, Oxfordshire Community Stroke Project; TOAST, Trial of Org 10172 in Acute Stroke Treatment.

*Supplementary figure S1*. The confusion matrices of classifiers. SVM, support vector machine; LR, logistic regression; RF, random forest.
